# Clinical characteristics from co-infection with avian influenza A H7N9 and *Mycoplasma pneumoniae*: a case report

**DOI:** 10.1186/s13256-018-1583-5

**Published:** 2018-03-15

**Authors:** Haili Luo, Shaohong Wang, Tongmei Yuan, Jingtao Liu, Ling Yao, Xianguo Pan, Xuemei Long, Juncheng Wu, Feng Shen

**Affiliations:** 1 Department of Critical Care Medicine, Affiliated Hospital of Guizhou Medical University, Guiyang, 550004 China; 2 Department of Critical Care Medicine, the Second Affiliated Hospital of Guizhou Medical University, Guiyang, 556000 China

**Keywords:** Avian influenza A, H7N9, *Mycoplasma pneumoniae*, Co-infection, Case report

## Abstract

**Background:**

More and more cases of human infections with avian influenza A H7N9 have been reported since it was first mentioned in 2013 in China, but concurrence of influenza A H7N9 with *Mycoplasma pneumoniae*, however, has never been described. Here, we reported the case of a woman co-infected by influenza A H7N9 and *Mycoplasma pneumoniae*, whose treatment process was a little bit longer and a little bit complicated as well.

**Case presentation:**

Our patient was an 80-year-old Chinese woman who presented with fever, cough, chest tightness, and shortness of breath. A computed tomography scan showed obvious infiltrations at lower parts of both lungs. Arterial blood gas analysis confirmed a severe respiratory failure (type I). Her sputum and throat swabs were checked for nucleic acid of influenza A and the result was positive for influenza A H7N9. She was diagnosed as having severe influenza A H7N9 and acute respiratory distress syndrome, and was admitted to an intensive care unit. She was given comprehensive treatment, including oseltamivir, methylprednisolone, immunoglobulin, gastric protection, and noninvasive mechanical ventilation. Her condition improved 4 days later. However, some symptoms exacerbated again 2 days later with ground-glass changes appearing in upper area of right lung and the titer of antibody to *Mycoplasma pneumoniae* rising from 1:80 to 1:640. She was reasonably considered to be infected with *Mycoplasma pneumoniae* as well, and azithromycin and moxifloxacin were added to her treatment. Oseltamivir was discontinued because of three consecutive negative results of nucleic acid for influenza A H7N9, but anti-*Mycoplasma* treatment was continued. Although her symptoms and abnormal changes on computed tomography scan slowly went away, she finally recovered from the mixed infection after a total of 33 days of management.

**Conclusion:**

In patients with confirmed influenza A H7N9 infection whose condition worsens again, especially with new infiltration or lung ground-glass infiltration, one should suspect infection by other pathogens such as *Mycoplasma pneumoniae*.

## Background

Since the case of human infection with avian influenza A H7N9 was first reported in 2013 in China [1], many more cases of this disease have emerged in this country [2, 3]. Human infection with avian influenza A H7N9 is a kind of acute respiratory disease which is caused by avian influenza A H7N9 virus, with more than 90% of the infected patients presenting with rapidly progressive pneumonia and 70% of them presenting with acute respiratory distress syndrome (ARDS). The mortality is as high as 30% [4].

Patients with influenza A H7N9 often have some underlying diseases including chronic obstructive pulmonary disease (COPD), hypertension, diabetes mellitus, and heart diseases [3], which make patients prone to infection by some other pathogens, such as bacteria. However, concurrence of influenza A H7N9 virus and *Mycoplasma pneumoniae* (MP) infection has never been described.

MP pneumonia is an atypical community-acquired pneumonia, which is most often mild and moderate, and its prognosis is usually good [5]. Here, we report a case with comorbidities of influenza A H7N9 and MP pneumonia, which made our patient’s treatment process a little bit longer.

Our patient is an 80-year-old retired worker, whose onset started with a fever, cough, abdominal distention, weakness, and anorexia. Then, chest tightness and short of breath followed. A computed tomography (CT) scan and arterial blood gas analysis confirmed the diagnosis of severe pneumonia and a respiratory failure (type I) for our patient. She was diagnosed with influenza A H7N9 by positive result of nucleic acid for influenza A H7N9 from sputum and throat swabs 10 days later and was transferred to an intensive care unit (ICU) in Second Affiliated Hospital of Guizhou Medical University, Kaili City, Guizhou Province, China. During her treatment process, she was confirmed to have an infection of MP.

Since this is the first report of influenza A H7N9 complicated with MP, and the anti-MP treatment in such cases might be different from that in MP infection alone, it is valuable to present our case report, sharing our experiences in this patient’s treatment. The main clinical characteristics of our patient and her treatment practice are reported below.

## Case presentation

**Fig. 1:**
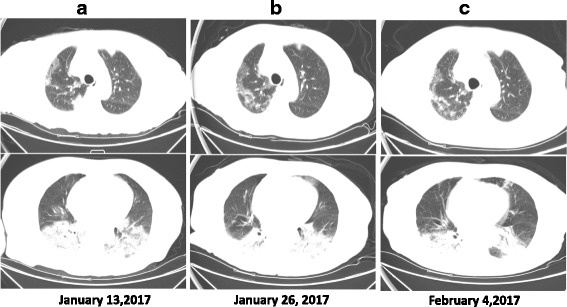
Evolution of lung imaging. **a** Ground-glass opacity in the upper field of the right lung and obvious consolidation and exudates in lower parts of both lungs. **b** Consolidation in low fields of both lungs decreased after 13 days of oseltamivir administration, but ground-glass opacity in upper area enlarged. **c** Ground-glass opacity of the upper area of the right lung alleviated with anti-*Mycoplasma pneumoniae* pneumonia therapy

An 80-year-old Chinese woman began to have fever, cough, abdominal distention, weakness, and anorexia on January 5, 2017. She had been to the local poultry market with her husband to buy a live chicken and had eaten chicken meat on January 2, 2017. She is a retired teacher, and she has three children, all of whom are healthy. She is optimistic and has healthy social relations. She currently lives with her husband. She went to see a doctor at a hospital in the southeast state of Guizhou Province on January 11, 2017. An examination showed a fever with body temperature of 38.3 °C, blood pressure of 130/78 mmHg, and pulse rate of 85 beats per minute (bpm). She was oriented and cooperative and in fine general condition. Her mental status was conscious and her neurological examination was normal. She does not smoke tobacco and does not consume alcohol. Her sputum and throat swabs were collected to test the nucleic acid of avian influenza A virus. Then, she was given some general anti-virus drugs. Fever persisted and she felt short of breath, so she went to the Second Affiliated Hospital of Guizhou Medical University on January 13, 2017. An examination revealed a highest body temperature of 39.5 °C, pulse rate of 83 bpm, respiratory rate of 25 to 28 breaths per minute, moist rales located at middle-lower fields of both lungs, blood oxygen saturation (SpO_2_) of 94% (oxygenation at 4 L/minute of oxygen with a nasal catheter), noninvasive blood pressure of 112/64 mmHg, white blood cell count of 3.46 × 10^9^/L (neutrophils, 69.4%; lymphocytes, 0.8 × 10^9^/L), oxygen arterial tension (PaO_2_) of 58.4 mmHg with fraction of inspired oxygen (FiO_2_ 0.41), and carbon dioxide arterial tension (PaCO_2_) of 31.8 mmHg. A CT scan showed obvious infiltrates in both lungs and some ground-glass opacity in the middle field of her right lung (Fig. 1a). In her past medical history, she had hypertension and COPD. Because of suspicion for contagious disease, she was admitted to the Department of Infectious Disease of our hospital and given antibiotics, oxygen therapy, and oseltamivir. On January 14, 2017, she was definitely diagnosed as having influenza A H7N9 by the positive results of nucleic acid testing and was separated from other patients. She was transferred to the ICU on the same day because of obvious dyspnea and shortness of breath. Her immediate examination on ICU admission showed oxygen pulse saturation of 65%, a body temperature of 38.5 °C, noninvasive blood pressure of 128/75 mmHg, a maximal respiratory rate of 30 to 35 breaths per minute, and she was conscious and cooperative. Her neurological examination was completely normal. Arterial blood gas result revealed a PH of 7.48, PaCO_2_ of 31 mmHg, PaO_2_ of 49.1 mmHg (FiO_2_ 0.41), and bicarbonate (HCO_3_^−^) of 22.8 mmol/l; biochemical results consisted of total protein of 57.38 g/l, proalbumin of 69 mg/l, potassium of 3.39 mmol/l, sodium of 129.36 mmol/l, creative kinase of 162.15 U/l, creative kinase–myocardial band (MB) of 8 U/l, lactate dehydrogenase of 420 U/l, brain natriuretic peptide (BNP) of 185 ng/l, and procalcitonin (PCT) of 0.056 μg/L. The titer of antibody to MP was 1:80. Liver and renal functions were almost normal. Urine analysis showed red blood cell of 8 cells/low-power field (LP) and white cell of 10 cells/LP.

The main diagnoses at ICU admission were severe pneumonia and ARDS.

After being delivered to the ICU, our patient was given comprehensive treatments, including antibiotics, intravenously administered methylprednisolone (120 mg/day), immunoglobulin (10 g/day), and gastric protection. Oseltamivir at 300 mg/day was orally administered for the first 6 days, then at 150 mg/day for the following 3 days. As she was conscious and very cooperative, she was given noninvasive mechanical ventilation (PHILIPS Respironics V60, Respironics California, Inc, USA). Her input of fluid volume was strictly limited and her hemodynamics were stable except for occasional slight fluctuations. After having been treated for 4 days, her respiratory symptoms began to alleviate, and oxygenation improved with PaO_2_ up to 66 mmHg (FiO_2_ 0.4), and respiratory rate decreasing to 24 to 28 breaths per minute. However, 2 days later, she began to cough with less sputum, and tachypnea and weakness were exacerbated. The titer of MP antibodies gradually increased from 1:80 to 1:160, and then arrived at 1:640 within 9 days. A CT scan on January 26, 2017 revealed an enlarged ground-glass infiltrate in the upper area of her right lung (Fig. 1b). She was reasonably considered to be over-infected with MP, and then orally administered azithromycin (0.5 g/day) and intravenously administered moxifloxacin (400 mg/day) were prescribed on January 23, 2017. At the same time, oseltamivir was discontinued because of three consecutive negative results of nucleic acid for influenza A H7N9. After 5 to 6 days of anti-MP treatment, her symptoms alleviated and the titer of the antibodies decreased. A gradual reabsorption of ground-glass infiltrates was seen in upper lobe of her right lung after 9 days of treatment against MP (Fig. 1c). She was discharged on February 5, 2017 with mild cough and shortness of breath on exercises.

Considering that she still had a mild cough and felt somewhat short of breath, especially on exercises, and because some infiltrates in both lungs remained, she was advised to continue taking azithromycin regularly after discharge. We kept a close follow-up on her treatment. On February 7, 2017, the titer of MP antibodies decreased to 1:80. On February 13, 2017, all her symptoms had disappeared after a continuous 22 days of treatment against MP. The follow-up at 6 months after her discharge from ICU revealed that she has kept very fit. Her clinical course is summarized in Table 1.

**Table 1:** Main clinical process of the first case co-infected by avian influenza A H7N9 and mycoplasma pneumoniae in Kaili, Guizhou province, China

Course	Date	Clinical major events
day 1	5 January, 2017	Fever, cough, abdominal distention, weakness, anorexia
day 7	11 January, 2017	Went to see doctor, and sputum and throat swabs were collected to be checked for the presence of avian influenza virus
day 9	13 January, 2017	Admitted to Second Affiliated Hospital of Guizhou Medical University, CT scan showed obvious consolidation and infiltration in bilateral lungs and ground-glass changes in upper area of right lung; and she was diagnosed with pneumoniae, Oseltamivir and meropenem were administered
day 10	14 January, 2017	Result of neucleic acid testing for H7N9 was positive and the patient was transferred to ICU because of illness deterioration and ARDS. Non-invasive MV. Titer of antibody to mycoplasma pneumoniae was 1:80;
day 14	18 January, 2017	CHest X-ray showed the infectious focus of bilateral lung partially absorbed.
day 15	19 January, 2017	Cough again with fewer sputum, other symptoms exacerbated again, increase of moist rale in upper area in right lung; Titer of antibody to mycoplasma pneumoniae rised to 1:160;
day 19	23 January, 2017	CT scan showed new focus appearing in upper aera while old infultration being absorbed. Titer of antibody to mycoplasma pneumoniae arrived to 1:640, and azithromycin and moxifloxacin were administered.
day 19	23 January, 2017	Oseltamivir was discontinued because of three continual examinations of neucleic acid for influenza A H7N9 changed to negative
day 22	26 January, 2017	CT scan shground-glass focus enlarged comparing to that at 12 days before.
day 25	29 January, 2017	The titer of the antibody decreased to 1:320.Patient’s symptoms were alleviated and moist rales reduced
day 32	4 February, 2017	CT scan revealed the right upper focus obviously absorbed. Moxifloxacin was discontinued while azithromycin was till continued.
day 33	5 February, 2017	Discharged from hospital.
day 35	7 February, 2017	Titier of antibody to M. pneumoniae decreased to 1:80.
day 41	13 February, 2017	All symptoms disappeared and azithromycin discontinued.

## Discussion

Our patient suffered from avian influenza A H7N9 maybe because of contact history of live birds and her condition exacerbated in a short time. In her treatment process, she was confirmed to be over-infected by MP, which has not been reported before. The anti-MP process in this patient was longer than usual because of her poor underlying condition.

Until now, human infection with avian influenza A H7N9 has been merely confirmed to be associated with exposure to poultry [6]. This was the case for our patient, who had been to a poultry market and had eaten chicken prior to the onset of illness. Surprisingly, her husband, who went to the market with her and had closer contact with the live poultry, remained healthy. This may be associated with his relatively perfect immune system and with no obvious underlying diseases. By contrast, his wife suffered from some underlying illnesses including hypertension and COPD, which made her more susceptible to infection from other pathogens such as avian influenza A H7N9 virus.

Clinical studies have demonstrated that a proportion of patients with influenza A or B are simultaneously infected by other pathogens such as bacteria and aspergilla [3, 7, 8] because of some risk factors such as COPD, use of steroids, and diabetes mellitus or even with a immunocompetent status [8]. But as far as we know, the clinical condition of co-infection caused by influenza A H7N9 and MP has not been described before. Although “atypical” pneumonia was recognized in the 1930s, MP was actually isolated from the sputum of a patient with “atypical” pneumonia in 1944 [9]. Early clinical observations indicated a relatively high prevalence of MP respiratory infection in younger children, especially among school-aged children from 5 to 15 years of age [10]. So far, there have been fewer reports of MP respiratory infection in older individuals.

Lung infection caused by MP can be diagnosed with culture of respiratory specimens and/or a fourfold rise in antibody titer to MP within 2 weeks [11]. In our patient, the MP antibody titer rose fourfold within 9 days, along with the exacerbation of some symptoms and appearance of some new ground-glass lung infiltrates. This supported our diagnosis of MP in this patient [11]. Since the incubation period of MP infection is often as long as 1 to 3 weeks [5], we speculate that MP may have already existed in our patient’s respiratory system at the time when she was infected with avian influenza A H7N9.

Although MP infections are generally mild, patients may develop severe and fulminant disease at any age [12]. This is especially the case in patients who simultaneously suffer from another disease such as avian influenza A H7N9, as observed in our patient.

The lack of a cell wall makes MP resistant to cell wall synthesis inhibitors such as β-lactam antibiotics. Effective drugs against MP include macrolides, tetracyclines, and fluoroquinolones [13]. Our patient was given azithromycin and moxifloxacin, and the ultimate result indicates that this therapeutic regimen was fortunately efficacious. It is generally recommended that the duration of treatment with azithromycin for community-acquired MP pneumonia is 7 to 14 days [14]. For our patient, azithromycin administration persisted for 22 days because of her poor underlying condition and her immunocompromised status.

It is noteworthy that although no fluoroquinolone resistance has been described so far in MP clinical isolates, more and more macrolide-resistant MP have been reported in recent years [14, 15]. If the clinical manifestations do not resolve in 72 hours with macrolides administration, it is recommended to change antibiotics to tetracycline (doxycycline and minocycline) or fluoroquinolones (moxifloxacin and levofloxacin) [15].

## Conclusion

In cases of confirmed influenza A H7N9, if the condition of the patient is not under initial treatment or if it even worsens with new ground-glass lung infiltrates, infection with another pathogen including MP must be suspected and sought.

## Abbreviations

ARDS: Acute respiratory distress syndrome
bpm: Beats per minute
COPD: Chronic obstructive pulmonary disease
CT: Computed tomography
FiO_2_: Fraction of inspired oxygen
ICU: Intensive care unit
LP: Low-power field
MP: *Mycoplasma pneumoniae*
PaCO_2_: Carbon dioxide arterial tension
PaO_2_: Oxygen arterial tension
